# Dynamics of mechanical waves in periodic grapheme nanoribbon assemblies

**DOI:** 10.1186/1556-276X-6-430

**Published:** 2011-06-17

**Authors:** Fabrizio Scarpa, Rajib Chowdhury, Kenneth Kam, Sondipon Adhikari, Massimo Ruzzene

**Affiliations:** 1 Advanced Composites Centre for Innovation and Science, University of Bristol, BS8 1TR Bristol, UK; 2 Multidisciplinary Nanotechnology Centre, Swansea University, SA2 8PP Swansea, UK; 3 School of Aerospace Engineering, Georgia Institute of Technology, Atlanta, GA 30332, USA

## Abstract

We simulate the natural frequencies and the acoustic wave propagation characteristics of graphene nanoribbons (GNRs) of the type (8,0) and (0,8) using an equivalent atomistic-continuum FE model previously developed by some of the authors, where the C-C bonds thickness and average equilibrium lengths during the dynamic loading are identified through the minimisation of the system Hamiltonian. A molecular mechanics model based on the UFF potential is used to benchmark the hybrid FE models developed. The acoustic wave dispersion characteristics of the GNRs are simulated using a Floquet-based wave technique used to predict the pass-stop bands of periodic mechanical structures. We show that the thickness and equilibrium lengths do depend on the specific vibration and dispersion mode considered, and that they are in general different from the classical constant values used in open literature (0.34 nm for thickness and 0.142 nm for equilibrium length). We also show the dependence of the wave dispersion characteristics versus the aspect ratio and edge configurations of the nanoribbons, with widening band-gaps that depend on the chirality of the configurations. The thickness, average equilibrium length and edge type have to be taken into account when nanoribbons are used to design nano-oscillators and novel types of mass sensors based on periodic arrangements of nanostructures.

**PACS **62.23.Kn · 62.25.Fg · 62.25.Jk

## Introduction

Graphene nanoribbons (GNRs) [1] have attracted a significant interest in the nanoelectronics community as possible replacements to silicon semiconductors, quasi-THz oscillators and quantum dots [2]. The electronic state of GNRs depend significantly on the edge structure. The zigzag layout provides the edge localized state with non-bonding molecular orbitals near the Fermi energy, with induced large changes in optical and electronic properties from quantization. DFT calculations and experimental measurements have shown that zigzag edge GNRs can show metallic or half-metallic behaviour (depending on the spin polarization in DFT simulations), while armchair nanoribbons are semiconducting with an energy gap decreasing with the increase of the GNR width [3-5]. GNRs have also been prototyped as photonics waveguides by Law et al. [6], and recently proposed for thermal phononics to control the reduction of thermal conductivity by Yosevich and Savin [7].

In this study, we describe the mechanical vibration natural frequencies and acoustic wave dispersion characteristics of graphene nanoribbons considered as periodic structures. In structural dynamics design, the wave propagation characteristics of periodic systems (both 1D and 2D) have been extensively used to tune the acoustic and vibrational signature of structures, materials and sensors [8-10], while at nanoscale level the periodicity of nanotubes array has also been used to develop nanophotonics crystals (see for example the study of Kempa and et al. [11]). Hod and Scuseria have also observed that the presence of a central mechanical load (or uniform inposed displacements) in bridged-bridged nanoribbons induces a significant electromechanical response in bending and torsional deformations [5]. We focus in this article on nanoribbon architectures of the type (8,0) and (0,8). While the results present in this manuscript are related to these specific nanoribbon topologies, the general algorith that we proposed can be readily extended to analyse more general graphene architectures. The nanoribbon models are developed using a hybrid atomistic continuum-Finite Element (FE) model (also called lattice [12]), in which the carbon-carbon (C-C) covalent bonds are represented by Timoshenko structural beams with equivalent mechanical properties (Young's modulus and Poisson's ratio) derived by the minimisation of the Hamiltonian of the structural system, or total potential energy for the static case [12-14]. It is worth to notice that the concept of the Hamiltonian of a system is not limited to problems associated to quantum mechanics, but it is also used in a large variety of variational problems related to the dynamics and stability of engineering and mechanical structures [15,16]. The equivalent mechanical properties for the *sp*^2 ^C-C bond are expressed in terms of the thickness of the bond itself. It is useful to reiterate that there is neither a physical thickness *per se *for the covalent bonds, nor for the carbon atoms involved in the bond. Nonetheless, when subjected to a mechanical static loading, the nanostructure tends to reach its equilibrium state corresponding to the minimum potential energy. The geometric and material configuration of the equivalent continuum mechanics structures used to represent the graphene (plates and/or shells) will be therefore be defined by the energy equilibrium conditions of the nanostructure, and cannot be ascribed as fixed. The length of the covalent bonds merits also some considerations. In finite size rectangular single layer graphene sheets (SLGS), the lengths of the C-C bonds at equilibrium after mechanical loading are unequal, ranging between 0.136 and 0.144 nm, and depend on the type of loading, size and boundary conditions [17,18], as well as the location on the SLGS itself (i.e. the edges [19]). This fact contrasts with the classical use of the fixed value of 0.142 nm at equilibrium considered in most mechanical simulations [20-23]. The variation of the thickness and the distributions of lengths at equilibrium is important factors to consider when computing the *homogenised *mechanical properties of the graphene, i.e. the equivalent mechanical performance of the graphene seen as a *continuum*. In this study, we will show that the thickness and the equilibrium length distributions assume some specific values in GNRs also when undergoing a mechanical resonant behaviour, both as a single nanostructure in free-free vibration conditions, and as periodic elements in a one-dimensional (1D) acoustic wave propagation case. However, the thickness and equilibrium lengths for the mechanical vibration case will be determined minimimsing the Hamiltonian of the system, rather that the total potential energy of the static loading case. Similar to the static in-plane and out-plane loading cases [12,13], those values can be different from the ones usually adopted in open literature. We will also show that the chirality of the GNRs (and their edge effects in nanoribbons with short widths) provides different acoustic wave dispersion properties, which should be taken into account when GNRs are considered for potential nanoelectromechanical systems (NEMS) applications.

## Modeling

### Atomistic-FE model

We use the atomistic-continuum equivalence model for the *sp*^2 ^carbon-carbon bonds to extract the equivalent isotropic mechanical properties (Young's modulus and Poisson's ratio) as a functions of the thickness *d *of the C-C bond [13,14]. The model is based on the equivalence between the harmonic potential provided by force models such as AMBER or linearised Morse, and the strain energies associated to out-of-plane torsional, axial and bending deformation of a deep shear Timoshenko beam:(1)

The first row of (1) corresponds to the equivalence between stretching and axial deformation mechanism (with *E*_Y _being the equivalent Young's modulus), while the second one equates the torsional deformation of the C-C bond with the pure shear deflection of the structural beam associated to an equivalent shear modulus *G*. Contrary with analogous approaches previously used [21,23], the term related to the in-plane rotation of the C-C bond (third row of 1) is equated to a bending strain energy associated to a deep shear beam model, rather than a flexural one, to take into account the shear deformation of the cross section. The shear correction term becomes necessary when beams assume aspect ratios lower than 10 [24], which is the case for the C-C bonds with average lengths and thickness presented in in open literature (see the article of Huang et al. [25]). For circular cross sections, the shear deformation constant can be expressed as [13]:(2)

In (2), *A*_s _= *A*/*F*_s _is the reduced cross section of the beam by the shear correction term *F*_s _[26]:(3)

The insertion of (2) and (3) in (1) leads to an nonlinear relation between the thickness *d *and the Poisson's ratio *ν *of the equivalent beam [13]:(4)

where(5)(6)

The values for the force constants for the AMBER model are *k_r _*= 6.52 × 10^-7 ^N·mm^-1^, *k_θ _*= 8.76 × 10^-10 ^N · nm · rad^-2 ^and *k_τ _*= 2.78 × 10^-10^N · nm^-1 ^· rad^-2^. The equivalent mechanical properties of the C-C bond can be determined performing a nonlinear optimisation of (1) using a Marquardt algorithm. The C-C bond can then be discretised as a single two-nodes three-dimensional Finite Element model beam with a 6 × 6 stiffness matrix [**K**]**_e _**described in [27], where the nodes represent the atoms. The mass matrix [**M**]**_e _**of the bond is represented through a lumped matrix approach [28]:(7)

where *m_c _*= 1.9943 × 10^-26^*kg*. The elemental matrices are then assembled in the usual Finite Element fashion as global stiffness and mass matrices [**K**] and [**M**], respectively, which can be subsequently used to formulate the undamped eigenvalue problem [29]:(8)

Equation 8 is solved using a classical Block Lanczos algorithm implemented in the commercial FE code ANSYS (Rel. 12). According to Equation 2-4, the natural frequencies *ω_i _*are, however, dependent on the thickness *d*. In the hybrid FE simulation, we consider also the variation of the average bond length *l *across the graphene sheet, a phenomenon observed in several models of SLGSs subjected to mechanical loading [13,17,19,30]. To identify a unique set of thickness and equilibrium lengths for a specific eigensolution, we minimise the Hamiltonian of the system [15]:(9)

where *T *and *U *are the kinetic and strain energies of the system, respectively. Using the mass-normalized normal modes [**Φ**] associated to the eigenvalue problem [29], the Hamiltonian (9) for each eigensolution *i *can be rewritten as:(10)

The 1D wave propagation analysis is carried out using a technique implemented by Tee et al. [10] and Aberg and Gudmundson [31]. Applying the Floquet conditions between the left and the right nodal degrees of freedom (DOFs) {**u**}*^L ^*and {**u**}*^R ^*one obtains:(11)

where -*π *≤ *k_x _*≤ *π *is the propagation constant within the first Brillouin zone [32]. The generalized DOFs of the system will be complex (real and imaginary part), while for traveling waves the propagation constant *k_x _*will be solely real [32]. Equation 11 can be, therefore, recast as:(12)

The real and imaginary parts of the domain in the FE representation are produced creating two superimposed meshes, linked by the boundary conditions [10,31] (12). For a given wave propagation constant *k_x_*, the resultant eigenvalue problem provides the frequency associated to the acoustic wave dispersion curve. Similar to the undamped eigenvalue problem, the minimisation of the Hamiltonian (10) is also carried out for the wave propagation case to identify the set of thickness and average bond length required for the eigenvalue solution.

### Molecular mechanics approach

The molecular mechanics (MM) simulations were performed with Gaussian [33], using the universal force field (UFF) developed by Rappe et al. [34]. Force-field-based simulations are convenient to represent the acoustic/mechanical dynamics behaviour, because they use explicit expressions for the potential energy surface of a molecule as a function of the atomic coordinates. The UFF is also well suited for dynamics simulations, allowing more accurate vibration measurements than many other force fields, which do not distinguish bond strengths. The UFF is a purely harmonic force field with a potential-energy expression of the form:(13)

The valence interactions consist of bond stretching (*E_R_*), which is a harmonic term and angular distortions. The angular distortions are the bond angle bending (*E_θ_*), described by a three-term Fourier cosine expansion, the dihedral angle torsion (*E_ϕ_*) and inversion terms (out-of-plane bending) (*E_ω_*). *E_ϕ _*and *E_ω _*are described by cosine-Fourier expansion terms. The non-bonded interactions consist of van der Waals (*E*_VDW_) and electrostatic (*E*_el_) terms. *E*_VDW _are described by a Lennard-Jones potential, while *E*_el _described by a Coulombic term. The functional form of the above energy terms is given as follows:(14)

Here *k*_1_, *k*_2_, *k*_3 _and *k*_4 _are force constants, *θ*_0 _is the natural bond angle, *D *is the van der Waals well depth, r* is the van der Waals length, *q_i _*is the net charge of an atom, *ε *is the dielectric constant and *r_ij _*is the distance between two atoms. In nanotubes, the atoms have no net charge, so the *E*_el _term is always zero. The torsion term, *E_ϕ _*, turns out to be of great importance. Detailed values of these parameters in Equation 14 can be found in Ref. [34]. Some of the authors have successfully used a similar MM approach to describe the mechanical vibrations of single-walled carbon nanotubes [35] and boron-nitride nanotubes [36]. Other molecular mechanics approaches have been successfully used to describe the structural mechanics aspects of SWCNTs and MWCNTs (see for example Sears and Batra [37]).

## Results and discussions

### Molecular mechanics and atomistic-FE models

**Figure 1:**
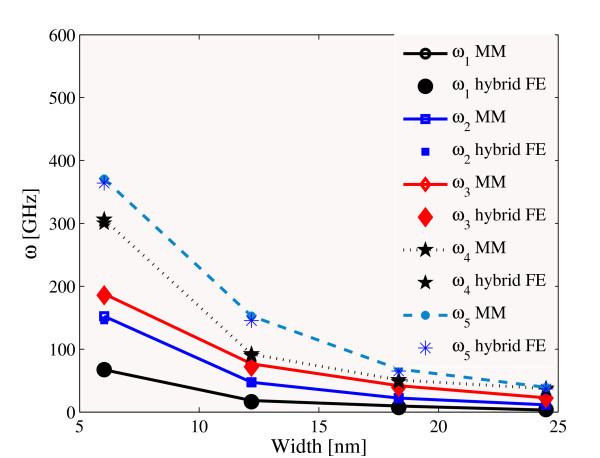
**Comparison between MM (full markers) and hybrid-FE (empty markers) natural frequencies for (8,0) SLGSs with different widths**.

Figure 1 shows the comparison between the MM simulations and the results from the hybrid FE models for a (8,0) nanoribbon at different lengths (6.03, 12.18, 18.34 and 24.49 nm). The equilibrium lengths are *l *= 0.142 nm for all cases considered. For the flexural modes the hybrid FE approach identifies a bond thickness *d *of 0.077 nm, with only a 3% difference from the analogous thickness value assocoated to the first torsional mode is considered. The identified thickness value compares well with the 0.074-0.099 nm found by some of the authors in uni-axial tensile loading cases related to single layer graphene sheets [13], with the 0.0734 nm in uni-axial stretching using first generation Brenner potential [25], and the 0.0894 nm identified by Kudin et al using *ab initio *techniques [38]. Gupta and Batra [39] find a thickness of 0.080 nm for the *ω*_11 _frequency of a fully clamped single layer graphene sheet (SLGS) with dimensions 3.23 nm × 2.18 nm, combining a MD simulation and results from the continuum elasticity of plates. It is worth to notice that these results are significantly different from the usual 0.34 nm inter-atomic layer distance adopted by the vast majority of the research community in nanomechanical simulations. The percentage difference between our MM and hybrid FE natural frequencies is on average around 3 for all the flexural modes. The torsional frequencies for the nanoribbons with the lowest aspect ratio provide a higher error (5%), suggesting that the assumption of equal in-plane and out-of-plane torsional stiffness with the AMBER model in Equation 1 leads to a slightly lower out-of-plane torsional stiffness of the nanoribbon.

### Wave propagation in bridged nanoribbons with different chirality

**Figure 2:**
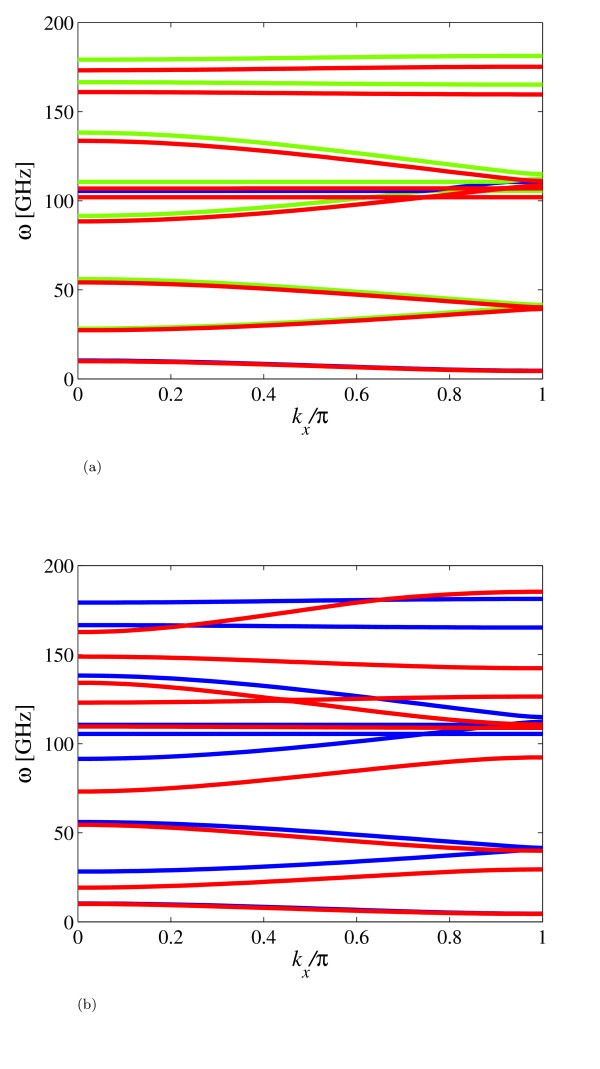
**Wave dispersion along the zigzag and armchair directions for a (8,0) GNR with length 15.854 nm**. (a) Continuous green line is referred to the Hamiltonian minimized versus *d*. Continuous red line is for the Hamiltonian minimized both for *d *and *l*. (b) Comparison of wave dispersions along the zigzag direction (continuous blue line) and armchair (continuous red line). The Hamiltonians are minimized for *d *only.

**Figure 3:**
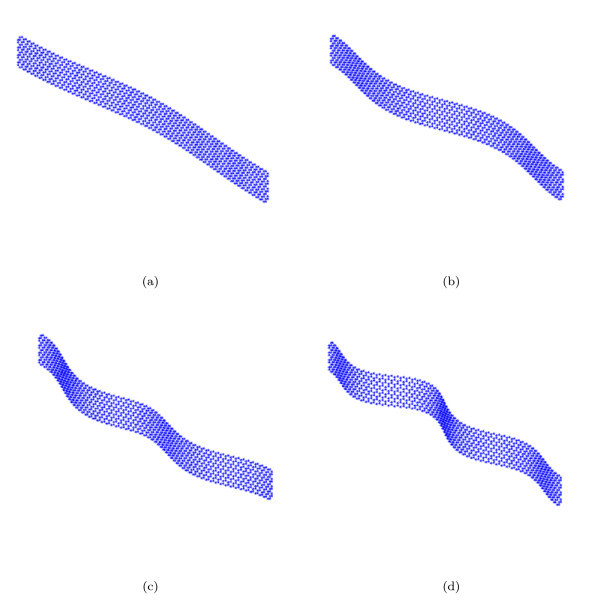
**Mode shapes (real parts) for a (8,0) GNR (length 15.854 nm) with propagation constant *k_x _*= *π*/4 along the zigzag direction**. (a) *ω*_1 _= 8.84 GHz; (b) *ω*_2 _= 29.35 GHz; (c) *ω*_3 _= 52.5 GHz; (d) *ω*_4_= 82.5 GHz.

The 1D wave propagation analysis has been carried out on (8,0) nanoribbons with a length of 15.854 nm along the zigzag direction, and 15.407 nm along the armchair direction for the (0,8) cases. The hybrid FE models have been subjected to simply supported (SS) conditions, clamping the relevant DOFs in the middle location of the ribbons, and allowing, therefore, to apply the relations (12) using a set of constraint equations. The wave dispersion characteristics for the propagation along the zigzag edge of the nanoribbons for the first Brillouin zone [32] are shown in Figure 2. The mode shapes associated to the first four pass-stop bands (Figure 3) are typical of periodic SS structural beams under bending deformation [40], while from our observations the out-of-plane torsional modes appear for the 5th and 6th wave dispersion characteristics.

**Figure 4:**
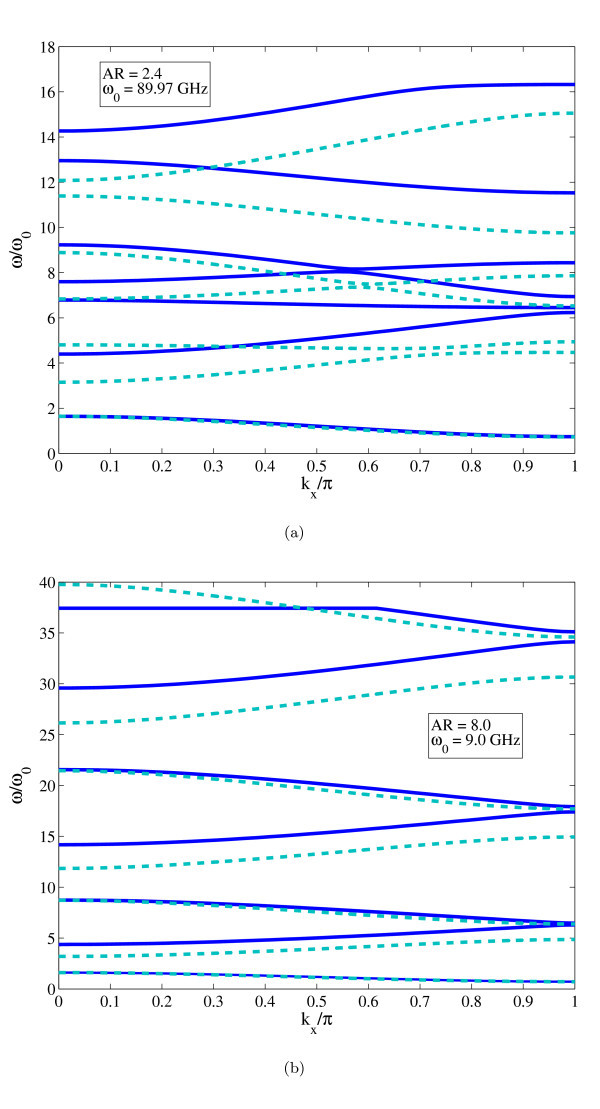
**Non-dimensional dispersion curves for zigzag (continuous lines) and armchair (dashed lines) (8,0) GNRs with different aspect ratios (AR)**. All the results are minimized for the thickness *d *only.

A more significant discrepancy between wave dispersion curves can be observed in Figure 2, when comparing the pass-stop band behaviour for the propagation along the zigzag and armchair directions. Only the first acoustic flexural wave dispersion characteristic is virtually unchanged, while for the other curves we observe a strong decrease in terms of magnitude, as well as mode inversion. The first stop band is significantly decreased by 25 GHz for *k_x _*= *π *- the armchair case gives a frequency drop of 39 GHz for the same propagation constant. Similar decreases in band gaps are observed for higher frequencies, while mode inversion (flexural to torsional) is observed for the armchair propagation around *k_x_*/*π *= 0.42, while for the armchair case the mode inversion is located around 0.8 *k_x_*/*π*. From the mechanical point of view, a possible explanation for this peculiar behaviour can be given considering the intrinsic anisotropy of the in-plane properties of finite size graphene sheets. Reddy et al. [17,41] have observed anisotropy ratios between 0.92 and 0.94 in almost square graphene sheets subjected to uni-axial loading, while similar orthotropic ratios have been identified also by Scarpa et al. [13]. The GNRs considered here have an aspect ratio close to 6, which induces the edges to provide a higher contribution to the homogenized mechanical properties due to Saint Venant effects [42]. A further confirmation of the effective in-plane mechanical anisotropy on the GNRs is apparent also from the non-dimensional dispersion curves shown in Figure 4. For that specific case, the GNRs have one side fixed (1.598 nm for the armchair, and 1.349 nm for the zigzag), with minimized thickness *d *equal to 0.074 and 0.077 nm and C-C bond equilibrium lengths of *l *= 0.142 nm for the armchair and zigzag cases, respectively. The dimensions of the nanoribbons are varied adjusting the aspect ratios (2.4 and 8), to obtain armchair and zigzag GNRs with similar dimensions. We have further nondimensionalised the dispersion curves using the values of the first dispersion relation (*ω*_0_) for the armchair configuration at *k_x _*= *π*/4. The GNR with an aspect ratio of 2.4 (Figure 4a) shows significant difference s in terms of dispersion characteristics between the armchair and the zigzag configurations, with a reduced band-gap of *Δ*(*ω*/*ω*_0_) equal to 3 for the armchair, against the value of 5 for the zigzag at the end of the first Brillouin zone (*k_x_*/*π *= 1). Between 4 <*ω*/*ω*_0 _*<*10, the wave dispersions appear to be composed by combinations of flexural plate-like modes with torsional components, with mode veering occurring between 0.45 *< k_x_*/*π *< 0.65. The zigzag-edged GNRs tend to show a narrowing of the nondimensional dispersion characteristics within the same *ω*/*ω*_0 _range considered. At higher non-dimensional wave dispersions, both armchair and zigzag nanoribbons tend to show beam-like dispersion characteristics [8,40,43]. The nanoribbons with higher aspect ratio (Figure 4b) show the pass-stop band behaviour typical of SS periodic structures made of Euler-Bernoulli beams [40]. However, while the first non-dimensional dispersion curve is identical, the following dispersion characteristics show a marked difference between zigzag and armchair configurations, with the zigzag GNRs having the highest *ω*/*ω*_0 _values. It is also worth of notice that while the zigzag configuration shows a dispersion curve provided by a torsional wave (straight line between 0 *< k_x_*/*π *< 0.62 at *ω*/*ω*_0 _= 37.4), the armchair GNR appears to be governed by flexural waves within the non-dimensional frequency interval considered. This type of behaviour suggests also that the specific morphology of the edges (combined with the small transversal dimensions of the GNRs) affect the acoustic wave propagation characteristics, both contributing to an overall mechanical anisotropy of the equivalent beams, as well as providing specific wave dispersion characteristics at higher frequencies. Moreover, the widening of the band gap observed in Figure 2 for the armchair configuration recalls some similarity to the variation of the energy gap of the electronic states noted in analogous armchair GNRs [3]. For a fixed width of 2.25 nm and and aspect ratio of 2.4 (i.e. 5.4 nm), the first pass-stop band at *k_x _*= 0 is located at 180 GHz. For the same fixed width but higher aspect ratio (8.0, corresponding to a transverse length of 18 nm), the same pass-stop band first frequency for *k_x _*= 0 is equal to 15 GHz, 12 times lower than the low aspect ratio case (Figure 4). Moreover, for the higher aspect ratio we observe a*Δω *= 18 GHz, while the lower aspect ratio provides a pass-stop band frequency interval *Δω *= 90 GHz, five times higher when compared for the armchair nanoribbons at AR = 2.4. Passing between lengths of 0.25 and 3 nm, Barone, Hod and Scuseria observe a decrease in energy gab by a factor of 3 for bare PBEs, and by 5 for bare HSEs [3]. When we consider the variation of the energy of the system proportional to the kinetic energy (and therefore approximately *Δω*^2^), ther ratio of the pass-stop bands for the armchair nanoribbons with different aspect ratios is compatible with the decrease of energy gap observed through DFT simulations [3].

## Conclusions

In this study, we have presented a new methodology to derive the mechanical structural dynamics characteristics and acoustic wave dispersion relations for graphene nanoribbons using an hybrid Finite Element approach. The technique, benchmarked against a Molecular Mechanics model, allows to identify the mechanical natural frequencies and associated modes shapes, as well as the pass-stop band acoustic characteristics of periodic arrays of GNRs.

The numerical results from the minimisation of the Hamiltonian in the hybrid FE method show that the commonly used value in nanomechanical simulations for the thickness (0.34 nm) is not adequate to represent the effective structural dynamics of the system. Thickness values identified through the minimisation of the Hamiltonian vary in a restricted range around 0.07 nm for the AMBER force model used in this study. We also observe a distribution of the C-C bond lengths corresponding to average values between 0.142 nm and 0.145 nm, after the minimisation for specific modes. However, the minimised thickness does not show any particular dependence over the type of mode shape considered. Only for pure torsional modes a small percentage variation from the baseline *d *= 0.074 nm value is observed.

We also show that graphene manoribbons exhibit a significant dependence of the acoustic wave propagation properties over the type of edge and aspect ratio, quite similarly to what observed for their electronic state. This feature suggests a possible combined electro-mechanical approach to design multifunctional waveguide-type band filters.

The use of periodic assemblies of graphene nanoribbons seems also a design feature that could lead to potential breakthroughs in terms of mass-sensors concepts, with enhanced selectivity provided by the periodic distribution of constraints and supports. The model proposed in this study allows to design and simulate these novel devices.

## Abbreviations

AR: aspect ratio; GNRs: graphene nanoribbons; MM: molecular mechanics; NEMS: nanoelectromechanical systems; 1D: one-dimensional; SS: simply supported; SLGS: single layer graphene sheet; SLGS: single layer graphene sheets; UFF: universal force field.

## Competing interests

The authors declare that they have no competing interests.

## Authors' contributions

FS carried out the lattice simulations for the graphene systems and wave propagation analysis for the (0,8) nanoribbons, and drafted the manuscript. KK performed the wave propagation simulations for the (8,0) nanoribbons. RC performed the MM simulations of the graphene sheets. SA conceived the comparison of the MM approach against the lattice model. MR helped to develop the 1D mechanical wave propagation model. All authors read and approved the final manuscript.
